# Hsa_circ_0021205 enhances lipolysis via regulating miR-195-5p/HSL axis and drives malignant progression of glioblastoma

**DOI:** 10.1038/s41420-024-01841-7

**Published:** 2024-02-10

**Authors:** Suwen Li, Jiaqi Yuan, Zhe Cheng, Yongdong Li, Shan Cheng, Xinglei Liu, Shilu Huang, Zhipeng Xu, Anyi Wu, Liang Liu, Jun Dong

**Affiliations:** 1 Department of Neurosurgery, the Second Affiliated Hospital of Soochow University, Suzhou, China; 2 Department of Neurosurgery, the Zhangjiagang Hospital of Traditional Chinese Medicine, Suzhou, China; 3 Department of Neurosurgery, the Second Affiliated Hospital of Bengbu Medical College, Bengbu, China; 4 Department of Neurosurgery, Affiliated Nanjing Brain Hospital, Nanjing Medical University, Nanjing, Jiangsu China

**Keywords:** CNS cancer, Cancer metabolism

## Abstract

Abnormal lipid metabolism is an essential hallmark of glioblastoma. Hormone sensitive lipase (HSL), an important rate-limiting enzyme contributed to lipolysis, which was involved in aberrant lipolysis of glioblastoma, however, its definite roles and the relevant regulatory pathway have not been fully elucidated. Our investigations disclosed high expression of HSL in glioblastoma. Knock-down of HSL restrained proliferation, migration, and invasion of glioblastoma cells while adding to FAs could significantly rescue the inhibitory effect of si-HSL on tumor cells. Overexpression of HSL further promoted tumor cell proliferation and invasion. Bioinformatics analysis and dual-luciferase reporter assay were performed to predict and verify the regulatory role of ncRNAs on HSL. Mechanistically, hsa_circ_0021205 regulated HSL expression by sponging miR-195-5p, which further promoted lipolysis and drove the malignant progression of glioblastoma. Besides, hsa_circ_0021205/miR-195-5p/HSL axis activated the epithelial-mesenchymal transition (EMT) signaling pathway. These findings suggested that hsa_circ_0021205 promoted tumorigenesis of glioblastoma through regulation of HSL, and targeting hsa_circ_0021205/miR-195-5p/HSL axis can serve as a promising new strategy against glioblastoma.

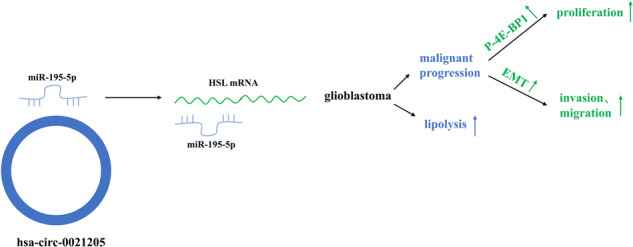

## Introduction

Glioblastoma is the most malignant primary brain tumor in adults [1], and the median survival of patients under standardized treatment is only 12-15 months [2], the 5-year survival rate is approximately 6.8% [3]. High infiltrative proliferation and invasion of glioblastoma limits complete surgical removal, blood-brain barrier (BBB) hinders the penetrating efficacy of most chemotherapeutic agents, resulting in the formation of such refractory tumors [4]. Some preclinical studies have shown that abnormal lipid metabolism was a fundamental feature of glioblastoma, which behaved higher intratumoral levels of fatty acids (FAs) and diverse phospholipids [5–7]. However, pathophysiological roles of lipid metabolism on glioblastoma development and progression, and the related regulatory pathways have not been fully elucidated.

Hormone-sensitive lipase (HSL), also known as lipase E, hormone-sensitive type (LIPE), constitutes a pivotal rate-limiting enzyme involved in the lipolysis of triacylglycerol (TAG). It acts synergistically with two other lipolytic enzymes, namely adipose triacylglyceride lipase (ATGL) and monoacylglycerol lipase (MAGL). Lipolysis is the processes of decomposing a TAG molecule into three FAs and one glycerol molecule, including ATGL hydrolyzing TAG to diacylglycerol (DAG), followed by HSL splitting DAG into monoglyceride (MAG), and MAGL decomposing MAG into FAs and glycerol finally [8].

Previous studies have shown that HSL played active roles in enhancing invasiveness of breast cancer cells through metabolic remodeling in adipocytes-rich tumor microenvironment [9, 10]. The direct interaction between HSL and DECR1 (2,4-Dienoyl-CoA reductase) augments lipolysis and facilitates the release of FAs, thereby fostering the migration and proliferation of cervical cancer cells [11]. The upregulated expression of HSL is significantly observed in the adipose tissue (AT) of cancer patients afflicted with cachexia [12], and inhibiting HSL expression preserves both AT and skeletal muscle mass in models of cancer-associated cachexia [13]. Therefore, aberrant upregulation of HSL expression was involved in cancer development.

CircRNAs played crucial roles in regulating tumor metabolic remodeling, thus promoting tumor progression [14]. CircWHSC1 was reported to regulate breast cancer progression by sponging miR-195-5p via FASN/AMPK/mTOR pathway to target on fatty acid synthesis [15]. CircRPL23A/miR-1233/ACAT2 axis suppressed malignant progression of renal clear cell carcinoma by restraining intracellular synthesis of cholesterol esters [16]. Many circRNAs have been reported in regulating glioblastoma development, however, their roles on lipid metabolic remodeling of tumor have not been fully elucidated.

In the current studies, based on differentially expressed HSL between glioblastoma and brain tissue, the molecular mechanisms by which HSL enhanced glioblastoma development, and the regulatory role of upstream non-coding RNAs in tumor lipolysis were investigated to screen out the potential new biomarkers against glioblastoma.

## Results

### HSL was highly expressed in glioblastoma

**Fig. 1:**
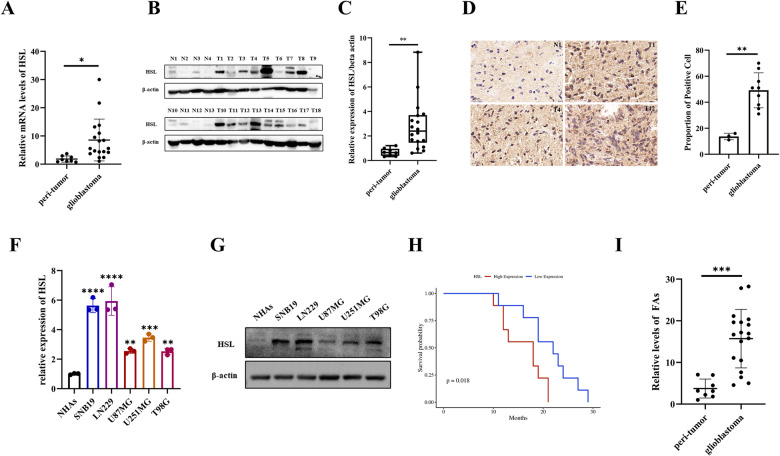
High expression of HSL in glioblastoma. **A** Expression of HSL in glioblastoma surgical specimen detected by qRT-PCR. **B**, **C** Expression of HSL in glioblastoma surgical specimen detected by Western blot and the box plot of HSL/beta-actin. **D**, **E** Immunohistochemical detection of HSL in representative para-tumor brain tissues and glioblastoma tissues. **F**, **G** HSL expression in human glioblastoma cell lines (SNB19, LN229, U87MG, U251MG, T98G) by qRT-PCR and Western blot. **H** Kaplan–Meier analysis of glioblastoma patients with relatively high or low expression of HSL. **I** Relative quantitation of FAs in 18 glioblastoma and 8 matched peri-tumor brain tissues. Data are expressed as mean ± SD. *p < 0.05, **p < 0.01, ***p < 0.001, ****p < 0.0001.

The expression status of HSL in 18 glioblastoma surgical specimens and 8 paired peri-tumor brain tissues were analyzed by qRT-PCR and Western blot, which showed that HSL expression in glioblastoma was higher than that of peri-tumor brain tissue (Fig. 1A–C). In addition, immunohistochemical staining (IHC) further confirmed high expression of HSL in clinical glioblastoma specimens (Fig. 1D, E). HSL expression in human glioblastoma cell lines (SNB19, LN229, U87MG, U251MG, T98G) was detected with qRT-PCR and Western blot as well, which disclosed that the expression of HSL in glioblastoma cell lines was significantly higher than that of normal human astrocytes (NHAs) (Fig. 1F, G). Kaplan–Meier analysis indicated that glioblastoma patients with high HSL expression had a poor outcome (Fig. 1H). The tissue content of FAs was also compared, which suggest that FAs content in glioblastoma clinical samples was higher than that of peri-tumor brain tissue (Fig. 1I). Therefore, high HSL expression in human glioblastoma was positively associated with high level maintaining of intratumoral FAs, which implied that HSL and FAs were involved in regulating glioblastoma development.

### HSL knockdown restrained the malignant phenotypes of glioblastoma cells, which can be rescued by supplement of FAs

**Fig. 2:**
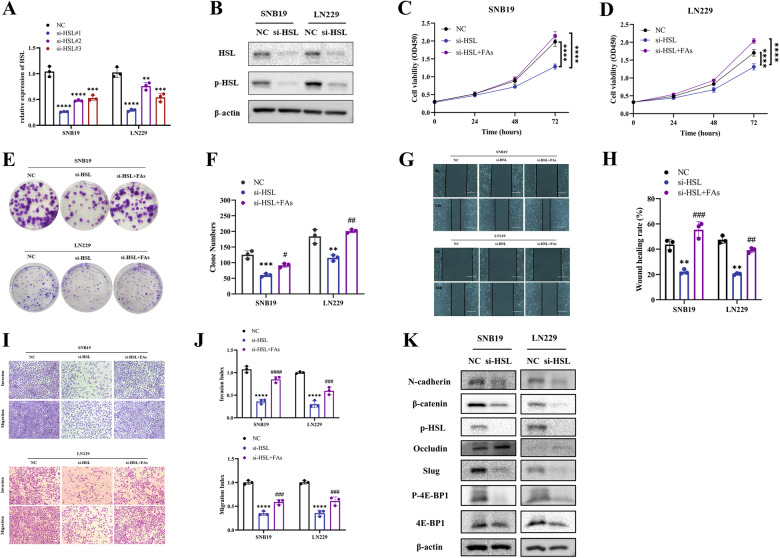
HSL knockdown restrained the malignant phenotypes of glioblastoma cells in vitro, and can be rescued by supplement of FAs. **A** Detection of cell transfection efficiency of HSL siRNAs by qRT-PCR. **B** Detection of cell transfection efficiency of HSL siRNA-1 by Western blot. **C**, **D** CCK8 assay was performed to detect cell viability in SNB19 and LN229 cells with NC, si-HSL transfection, or si-HSL supplemented with 10 μM FAs, respectively. **E**, **F** Colony formation assay was performed to evaluate the effect of HSL knockdown or si-HSL supplemented with 10 μM FAs on cell proliferation ability. **G**, **H** Wound healing assay was conducted to evaluate changes of cell migration ability of SNB19 and LN229 cells treated with NC, si-HSL or si-HSL supplemented with 10 μM FAs, respectively (bar = 400 µm). **I**, **J** Invasion and migration capabilities were determined by transwell assay in SNB19 and LN229 cells with NC, si-HSL or si-HSL supplemented with 10 μM FAs. **K** The protein expression of N-cadherin, Slug, β-catenin, Occludin, 4E-BP1 and p-4E-BP1 were analyzed by Western blot in SNB19 and LN229 cells transfected with NC or si-HSL. Data are expressed as mean ± SD. *p < 0.05, **p < 0.01, ***p < 0.001, ****p < 0.0001.

To further investigate the role of HSL in regulating glioblastoma cells proliferation, HSL-downregulating tumor cell lines (transfection of siRNAs into both SNB19 and LN229 cells) were established (Fig. 2A), and the designed interfering chain si‐HSL-1 showed the highest interference efficiency and was selected for subsequent experiment (Fig. 2B). CCK-8 assay and colony formation assay confirmed that HSL knocking-down significantly reduced the proliferation of SNB19 and LN229 cells, and can be reversed with supplementation of 10 μM FAs (Fig. 2C–F). Transwell and wound healing experiments further disclosed that knocking-down of HSL inhibited the invasion and migration of SNB19 and LN229 cells, which can be reduced by addition of 10 μM FAs (Fig. 2G–J). Besides, protein level of N-cadherin, Slug, β-catenin and p-4E-BP1 was positively correlated with HSL expression, while Occludin was negatively associated with HSL expression (Fig. 2K), suggesting downregulation of HSL weakened EMT processes of glioblastoma.

### HSL overexpression promoted the malignant phenotypes of glioblastoma cells in vitro

**Fig. 3:**
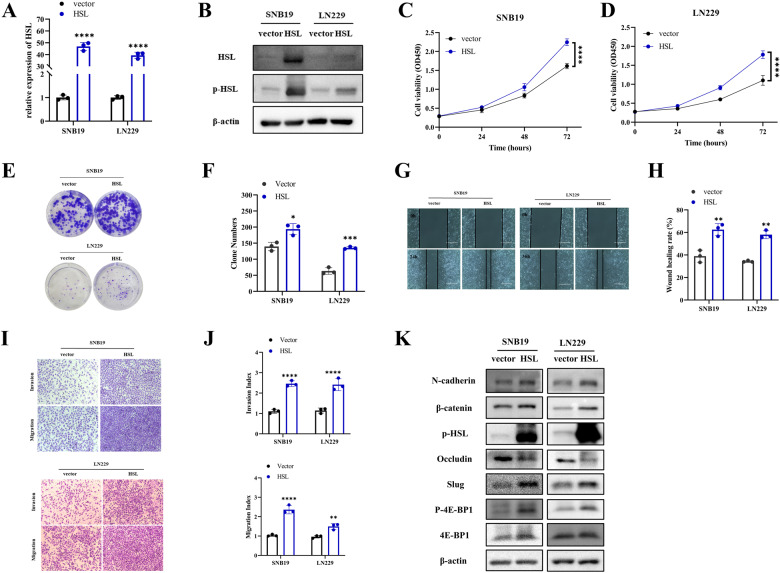
HSL overexpression promoted the malignant phenotypes of glioblastoma cells in vitro. **A**, **B** HSL expression in SNB19 and LN229 cells transfected with either empty or HSL overexpression vector were detected by qRT-PCR and Western blot. **C**, **D** CCK8 assay was utilized to compare cell proliferation of HSL overexpressed SNB19 or LN229 cells. **E**, **F** Colony formation assay was performed to evaluate the effect of HSL overexpression on cell proliferation ability. **G**, **H** Effect of HSL overexpression on SNB19 and LN229 cells migration evaluated by wound healing assay (bar = 400 µm). **I**, **J** Invasion and migration capabilities were examined by transwell assay in both SNB19 and LN229 cells with or without HSL overexpression. **K** The protein expression of N-cadherin, Slug, β-catenin, Occludin, 4E-BP1 and p-4E-BP1 were analyzed by Western blot in SNB19 and LN229 cells transfected with the empty vector or HSL-overexpression plasmids. Data are expressed as mean ± SD. *p < 0.05, **p < 0.01, ***p < 0.001, ****p < 0.0001.

The plasmids carrying empty vector, or HSL overexpression vectors were transfected into SNB19 and LN229 cells, respectively, and transfection efficiency was verified by qRT-PCR and Western blot (Fig. 3A, B). CCK-8 and colony formation assay showed that HSL overexpression obviously enhanced proliferation of SNB19 and LN229 cells (Fig. 3C, F). Wound healing assay and Transwell assay disclosed that HSL overexpression enhanced invasion and migration of SNB19 and LN229 cells (Fig. 3G–J). Western blot showed that HSL overexpression decreased Occludin expression, while increased expression of N-cadherin, Slug, β-catenin and p-4E-BP1 (Fig. 3K). In general, up-regulation of HSL expression enhanced the proliferation, invasion and migration of glioblastoma cells in vitro, as well as promoting glioblastoma EMT.

### MiR-195-5p negatively regulated HSL expression in glioblastoma cells

**Fig. 4:**
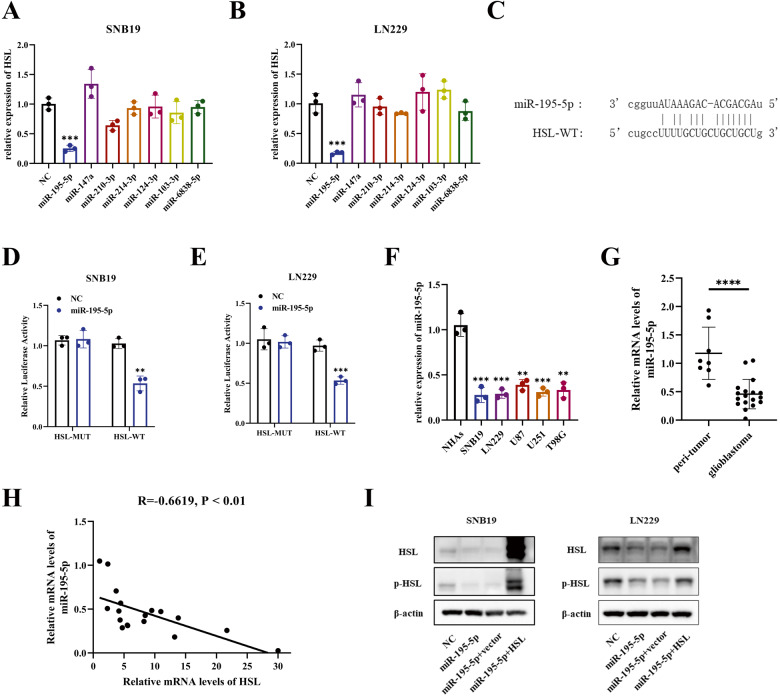
MiR-195-5p negatively regulated HSL expression in glioblastoma cells. **A**, **B** After overexpression of 7 miRNAs predicted by Starbase website, qRT-PCR was performed to evaluate the relative expression of HSL. **C** The binding sites between miR-195-5p and HSL predicted by Starbase. **D**, **E** miR-195-5p targets at HSL verified by dual-luciferase reporter assay. **F** The expression of miR-195-5p in glioblastoma cell lines (SNB19, LN229, U87MG, U251MG, T98G) by qRT-PCR. **G** miR-195-5p expression in 18 clinical glioblastoma specimens and 8 peri-tumor brain tissues by qRT-PCR. **H** Correlation analysis on the relationship between relative mRNA levels of miR-195-5p and HSL in 18 glioblastoma specimens. **I** The protein expression of HSL after transfection with NC, miR-195-5p mimics, miR-195-5p mimics+vector, or miR-195-5p mimics+HSL, respectively. Data are expressed as mean ± SD. *p < 0.05, **p < 0.01, ***p < 0.001, ****p < 0.0001.

For miRNAs play crucial roles in regulating lipid metabolisms, to explore whether miRNAs regulate HSL expression, online bioinformatic analysis was performed with StarBase v3.0 (http://starbase.sysu.edu.cn/) to predict miRNAs which combined with HSL. According to the specificity of binding site in UTRs and the number of databases in which the combination of HSL and miRNA can be predicted, altogether 7 miRNAs were predicted and screened out, which were most likely to combine with HSL. Then these miRNAs mimics were transfected into glioblastoma cells, and their impacts on HSL expression were evaluated, which disclosed that overexpression of miR-195-5p in SNB19 and LN229 cells can down-regulate HSL expression (Fig. 4A, B). The binding sites of HSL 3′-UTR and miR-195-5p were predicted by StarBase (Fig. 4C). The dual-luciferase report experiment showed that the mimics of miR-195-5p reduced the luciferase activity of HSL-WT, but failed to affect the mutant, indicating that miR-195-5p can directly bind to the target site of HSL-WT in SNB19 and LN229 cells (Fig. 4D, E). Quantitative RT-PCR verified low expression of miR-195-5p in both glioblastoma cell lines (SNB19, LN229, U87MG, U251MG, T98G) and glioblastoma clinical samples (Fig. 4F, G). Correlation analysis showed that the expression level of miR-195-5p was negatively correlated with the expression level of HSL in 18 clinical glioblastoma samples (Fig. 4H).In addition, the protein level of HSL and phospho-HSL (p-HSL) in glioblastoma cells was evaluated after transfection with NC, miR-195-5p mimics, miR-195-5p mimics+vector, or miR-195-5p mimics+HSL, respectively, which disclosed that the expression of HSL decreased after transfection of miR-195-5p mimics, and HSL overexpression reversed this effect partially (Fig. 4I).

### MiR-195-5p/HSL axis accelerated glioblastoma malignant progression in vitro

**Fig. 5:**
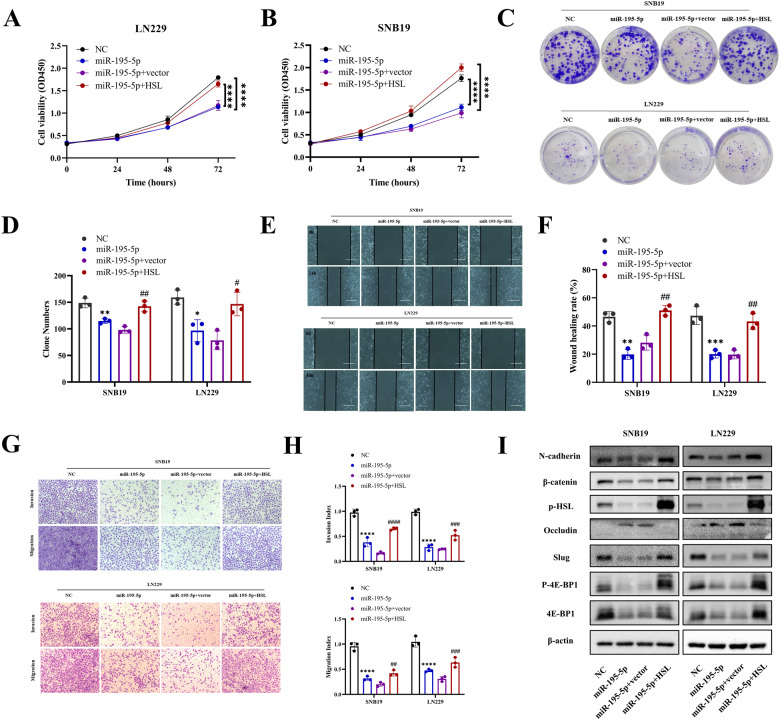
MiR-195-5p/HSL axis accelerated malignant progression of glioblastoma in vitro. **A**, **B** CCK8 assay, (**C**, **D**) Colony formation, (**E**, **F**) Wound healing assay (bar = 400 µm), (**G**, **H**) Invasion and migration assays were performed and quantitative analysis on SNB19 and LN229 cells transfected with NC, miR-195-5p mimics, miR-195-5p mimics+vector, or miR-195-5p mimics +HSL, respectively. **I** The protein levels of N-cadherin, Slug, β-catenin, Occludin, 4E-BP1 and p-4E-BP1 were analyzed by Western blot in SNB19 and LN229 cells transfected with NC, miR-195-5p mimics, miR-195-5p mimics+vector, or miR-195-5p mimics+HSL, respectively. Data are expressed as mean ± SD. *p < 0.05, **p < 0.01, ***p < 0.001, ****p < 0.0001.

To confirm the role of miR-195-5p in HSL on regulating malignant phenotypes of glioblastoma, CCK8 assay and colony formation assay were performed, which showed that combined overexpression of HSL and miR-195-5p can reverse the inhibition effect of miR-195-5p overexpression alone on proliferation of both SNB19 and LN229 cells (Fig. 5A, D). Transwell and wound healing assays disclosed that overexpression of HSL reversed the inhibition effects on invasion and migration of glioblastoma cells mediated by miR-195-5p (Fig. 5E–H). Besides, decreased level of N-cadherin, Slug, β-catenin and p-4E-BP1 expression can be observed in Western blot after miR-195-5p overexpression in glioblastoma cells, with increased Occludin expression level. Besides, in HSL-overexpression rescued glioblastoma cells, partially upregulation of Occludin and downregulation of N-cadherin, Slug, β-catenin and p-4E-BP1 can be observed (Fig. 5I). These findings further confirmed that miR-195-5p played a regulatory role in malignant progression of glioblastoma via inhibting HSL expression.

### Hsa_circ_0021205 sponged miR-195-5p to regulate HSL expression

**Fig. 6:**
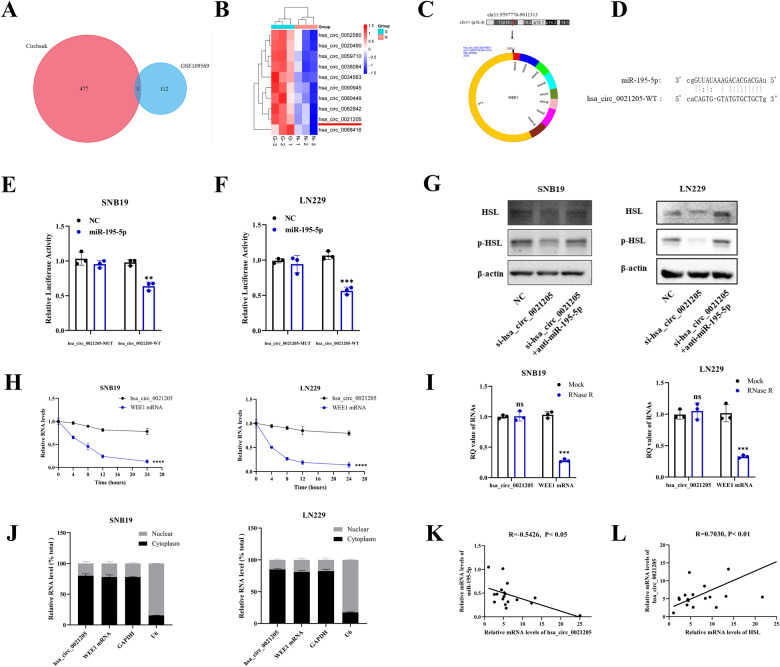
Hsa_circ_0021205 sponged miR-195-5p to regulate HSL expression. **A** Venn diagram of the overlapping target circRNAs of miR-195-5p predicted by Circbank and GSE109569 datasets. **B** Relative expression of hsa_circ_0021205 in GSE109569 datasets. **C** The location and illustration of hsa_circ_0021205. **D** Predicted binding sites between miR-195-5p and hsa_circ_0021205. **E**, **F** Dual-luciferase reporter assay was performed to verify the associative relations between hsa_circ_0021205 and miR-195-5p in SNB19 and LN229 cells. **G** Protein expression of HSL in SNB19 and LN229 cells after transfection with NC, si-hsa_circ_0021205, or si-hsa_circ_0021205+anti-miR-195-5p, respectively. **H** Relative mRNA expression of hsa_circ_0021205 and WEE1 after treatment with actinomycin D at the indicated time points. **I** RQ values of hsa_circ_0021205 and WEE1 mRNA in SNB19 and LN229 cells under RNase R treatment. **J** Nucleocytoplasmic fractionation revealed the subcellular localization of hsa_circ_0021205 in SNB19 and LN229 cells. **K** Correlation analysis on the relationship between relative mRNA levels of miR-195-5p and hsa_circ_0021205 in 18 glioblastoma specimens. **L** Correlation analysis on the relationship between relative mRNA levels of hsa_circ_0021205 and HSL in 18 glioblastoma specimens. Data are expressed as mean ± SD. *p < 0.05, **p < 0.01, ***p < 0.001, ****p < 0.0001.

CircRNAs can regulate lipid metabolism either by combining with their corresponding miRNAs, or direct interacting with their downstream target proteins. For further exploring the potential function of circRNA on miR-195-5p/HSL axis in triglyceride catabolism of glioblastoma, bioinformatic analysis of GSE109569 datasets in GEO database (including 3 glioblastoma clinical samples vs. 3 para-tumor brain samples) was applied, and genes with *P* < 0.05 and |Log2 (fold change)| >1 were considered to have significant changes. In addition, potential circRNAs binding with miR-195-5p were predicted through Circbank (http://www.circbank.cn/). Through the intersection of two datasets, hsa_circ_0021205 was screened out (Fig. 6A), and expression of hsa_circ_0021205 in GSE109569 dataset was analyzed (Fig. 6B). According to circPrimer 2.0 (https://www.bio-inf.cn/), hsa_circ_0021205 is located on chromosome 11 (chr11:9597776-9611313), produced by cyclization of exon 3-11 (spliced length: 2323) of host gene WEE1 (Fig. 6C). Circbank also predicted the binding sites of miR-195-5p and hsa_circ_0021205 (Fig. 6D). To further clarify the possibility of hsa_circ_0021205 binding to miR-195-5p, dual-luciferase report assay was performed in SNB19 and LN229 cells, which indicated that overexpression of miR-195-5p reduced the luciferase activity of hsa_circ_0021205-WT, but had no effect on hsa_circ_0021205-MUT (Fig. 6E, F). SNB19 and LN229 cells were transfected with NC, si-hsa_circ_0021205, or si-hsa_circ_0021205+anti-miR-195-5p, respectively, then the expression level of HSL in these cells was detected by Western blot, which showed that si-hsa_circ_0021205 decreased HSL level, while HSL overexpression reversed this effect (Fig. 6G). The transcriptional inhibition test of actinomycin D was performed, which showed that hsa_circ_0021205 had a longer half-life period than WEE1 mRNA (Fig. 6H). After treatment with RNase R, the level of linear WEE1 mRNA decreased sharply, while hsa_circ_0021205 mRNA level remained stable (Fig. 6I). Nucleocytoplasmic separation experiment showed that hsa_circ_0021205 was mainly located in the cytoplasm of glioblastoma cells (Fig. 6J). Correlation analysis showed that the expression level of hsa_circ_0021205 was negatively correlated with the expression level of miR-195-5p in 18 clinical glioblastoma samples (Fig. 6K), while positively correlated with the expression level of HSL (Fig. 6L). Therefore, these findings documented that hsa_circ_0021205 was a stable circRNA closely related to miR-195-5p and HSL.

### Hsa_circ_0021205 knockdown restrained malignant phenotypes of glioblastoma cells, which can be rescued by supplement of FAs or co-transfection with anti-miR-195-5p

**Fig. 7:**
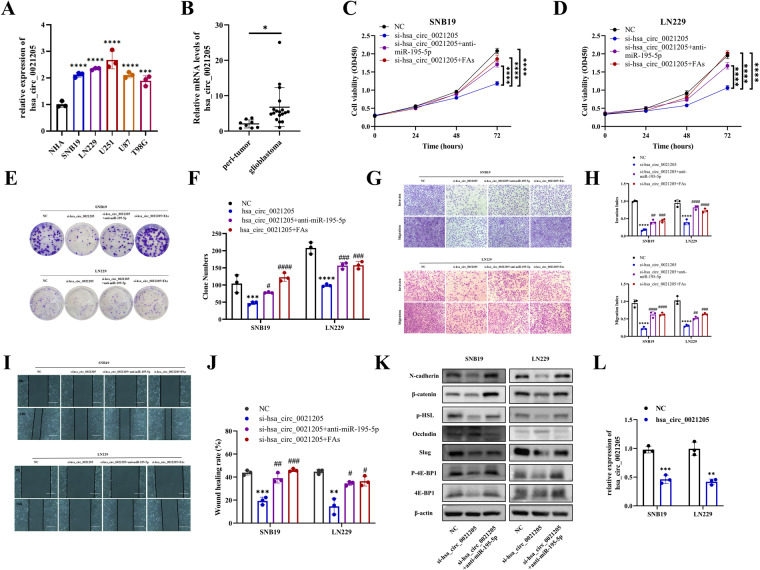
Hsa_circ_0021205 knockdown restrained the malignant phenotypes of glioblastoma cells in vitro, which can be rescued by either supplement of FAs or co-transfection with anti-miR-195-5p. **A**, **B** Expression of hsa_circ_0021205 in glioblastoma cell lines (SNB19, LN229, U87MG, U251MG, T98G) and clinical glioblastoma specimens detected by qRT-PCR. **C**, **D** CCK8 assay, (**E**, **F**) Colony formation, (**G**, **H**) Invasion and migration assays, and (**I**, **J**) Wound healing assay (bar = 400 µm) were performed and quantitatively analyzed in SNB19 and LN229 cells transfected with NC, si-hsa_circ_0021205, si-hsa_circ_0021205+anti-miR-195-5p, respectively. **K** The protein levels of N-cadherin, Slug, β-catenin, Occludin, 4E-BP1 and p-4E-BP1 were detected by Western blot in SNB19 and LN229 cells transfected with NC, si-hsa_circ_0021205, or si-hsa_circ_0021205+anti-miR-195-5p, respectively. **L** Transfection efficiency of si-hsa_circ_0021205 in SNB19 and LN229 cells detected by qRT-PCR. Data are expressed as mean ± SD. *p < 0.05, **p < 0.01, ***p < 0.001, ****p < 0.0001.

To further verify hsa_circ_0021205 level in glioblastoma, qRT-PCR was conducted, which showed higher expression of hsa_circ_0021205 in glioblastoma cell lines (SNB19, LN229, U87MG, U251MG, T98G)(Fig. 7A), and clinical specimens of tumor tissue (Fig. 7B). CCK8 assay and colony formation assay showed that both anti-miR-195-5p transfection or 10μm FAs supplementation in culture medium can reverse the inhibition effect of hsa_circ_0021205 knockdown on proliferation of SNB19 and LN229 cells (Fig. 7C–F). Transwell and wound healing assay also disclosed inhibition effect of hsa_circ_0021205 knockdown on invasion and migration abilities of glioblastoma cells (Fig. 7G–J). Western blot was applied to evaluate expression of N-cadherin, Slug, β-catenin and p-4E-BP1, which disclosed obviously reducing of these proteins in hsa_circ_0021205 knock-down glioblastoma cells, while Occludin level elevated. Besides, upregulation of Occludin and downregulation of N-cadherin, Slug, β-catenin and p-4E-BP1 were observed in the anti-miR-195-5p transfection rescued cells (Fig. 7K). To evaluate the function of hsa_circ_0021205 in glioblastoma cells, SNB19 and LN229 cells were transfected with si-hsa_circ_0021205 (Fig. 7L). These data further confirmed that hsa_circ_0021205 was crucial in malignant progression of glioblastoma by regulating miR-195-5p expression in vitro.

### Hsa_circ_0021205/miR-195-5p/HSL axis promoted abnormal lipolysis of glioblastoma cells

**Fig. 8:**
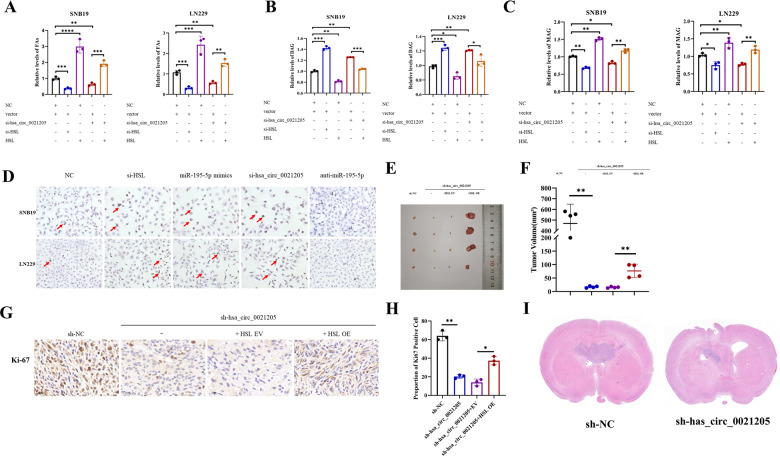
Hsa_circ_0021205/miR-195-5p/HSL axis promoted abnormal lipolysis in vitro and accelerates glioblastoma proliferation in vivo. **A** Relative intracellular FAs levels in SNB19 and LN229 cells after transfection. **B**, **C** Relative intracellular DAG and MAG levels in SNB19 and LN229 cells detected by ELISA after transfection. **D** Oil red O staining was conducted to stain intracellular neutral fat of SNB19 and LN229 cells in vitro. **E**, **F** In vivo evaluation of glioblastoma growth in subcutaneous tumor model. SNB19 cells with sh-NC, sh-hsa_circ_0021205, sh-hsa_circ_0021205 + HSL-EV, or sh-hsa_circ_0021205 + HSL-OE, respectively, were inoculated in the right subcutaneous axillary region of BALB/c nude mice. **G**, **H** Ki-67 positive cells were counted in the sections of xenografts by immunohistochemical staining. **I** Representative intracranial tumor xenografts of HE staining images are shown. Data are expressed as mean ± SD. *p < 0.05, **p < 0.01, ***p < 0.001, ****p < 0.0001.

The effect of hsa_circ_0021205 on lipolysis was investigated in glioblastoma cells. Intracellular FAs and MAG levels decreased following hsa_circ_0021205 knockdown in SNB19 and LN229 cells (Fig. 8A, C), while elevations in DAG levels can be observed (Fig. 8B). Simultaneous overexpression of HSL can reverse partially of this phenomenon by hsa_circ_0021205 knockdown. Oil red O staining showed that intracellular fat droplets increased significantly after transfection with si-hsa_circ_0021205, si-HSL or miR-195-5p mimics, while anti-miR-195-5p resulted in decreasing of fat droplets (Fig. 8D), suggesting hsa_circ_0021205/miR-195-5p/HSL axis promoted abnormal lipolysis and accelerated glioblastoma proliferation.

### Hsa_circ_0021205 accelerated glioblastoma proliferation in vivo

In order to study the roles of hsa_circ_0021205 and HSL in malignant progression of glioblastoma in vivo, SNB19 cells with stable sh-hsa_circ_0021205 transfection were established, as well as SNB19 cells with stable overexpression of HSL. Subcutaneous xenografting was applied, which disclosed that hsa_circ_0021205 downregulating significantly decreased xenografts size, whereas HSL overexpression can partially weakened the inhibition effect of hsa_circ_0021205 knockdown on tumor growth (Fig. 8E, F). IHC staining was performed to detect Ki67 expression in sections of xenografts, which showed a lower expression of Ki67 in hsa_circ_0021205 knocking-down tumors. In the HSL overexpression rescued SNB19 xenografts, Ki67 expression increased obviously (Fig. 8G, H). Intracranial orthotopic xenograft of SNB19 cells verified that hsa_circ_0021205 downregulating decreased xenografts size (Fig. 8I).

## Discussion

Although persistent achievements are still ongoing, glioblastoma is still a stubborn type of intractable adult brain tumor. The capacities of glioblastoma of strong invasiveness and infinite proliferation are largely due to its characteristics of metabolic reprogramming. As vital nutrients, FAs can promote glioblastoma growth, they play indispensable roles on both tumor progression and invasion [17, 18]. The triglyceride lipolysis pathway is a crucial pathway for supplying FAs to tumor cells, and the corresponding rate limiting enzyme HSL was directly relevant to various metabolic disorders, such as obesity, insulin resistance, cancer progression and cachexia, etc [19]. HSL was involved in accelerating malignant progression of breast cancer [9, 10] and cervical cancer [11]. However, whether HSL has the potential to promote glioblastoma development still needs further investigations. The current studies confirmed upregulation of HSL in both cellular and tissue level of glioblastoma. Enhancing HSL expression can promote proliferation, invasion, and migration of glioblastoma both in vitro and in vivo.

As a neutral lipase, HSL can recognize multiple lipid substrates and hydrolyze TAG, DAG, as well as MAG [20–23]. Besides, the binding affinity of HSL on DAG is about ten times higher than that of TAG, indicating that HSL mainly acts as a DAG hydrolase during lipolysis processes [24, 25]. Our results showed that upregulated HSL levels lead to a decline in intracellular DAG levels in glioblastoma cells, while FAs and MAG increase, with a more significant rise in FA levels. Conversely, downregulation of HSL produces the opposite effect, increasing intracellular DAG levels while decreasing MAG and FAs. For FAs can promote cancer cells proliferation through enhancing cellular membrane formation, increasing energy storage, and promoting the production of signaling molecules [17, 18], the current studies disclosed increased FAs content due to HSL upregulation can promote glioblastoma development as well. Knockdown of HSL impaired proliferation, invasion and migration abilities of glioblastoma cells, which can be reversed by adding to FAs.

The processes of tumor invasiveness were accompanied by complex cellular regulatory pathways, especially epithelial–mesenchymal transition (EMT), characterized by loss of intercellular adhesion (decreased expression of Occludin) and acquisition of mesenchymal features (increased expression of N-cadherin), under the regulation of EMT related transcription factors (EMT-TF) [26–28]. Slug was a zinc finger transcription factor involved in promoting EMT [27], β-catenin (core effector of the classic Wnt pathway) combined with p120-catenin and E-cadherin on the cell membrane to form complexes to connect to cytoskeleton, thus maintaining epithelial polarity and integrity [29]. Our studies revealed that downregulating HSL can decrease the expression of N-cadherin, β-catenin and Slug, and increase the expression of Occludin, while upregulation of HSL resulted in the opposite effects, indicating the possibility that HSL can promote invasion and migration of glioblastoma through EMT related pathway. Phosphorylation of EIF4E-binding protein 1 (p-4E-BP1, downstream effector of mTORC1) can promote cancer progression [30], we also found that downregulating HSL can restrain the expression of p-4E-BP1 obviously, which was consistent with the recent findings that high level of p-4E-BP1 was associated with poor prognosis in breast, ovarian, and prostate cancers [30], and suppressing p-4E-BP1 expression significantly inhibited cancer cells growth in vivo [31, 32].

Non-coding RNAs (ncRNAs) played significant roles in regulating malignant progression of tumors by mediating enzymes related to lipid metabolism, suggesting that ncRNAs have great potentials to serve as regulatory factors in lipid metabolism of tumors [33, 34]. MiRNAs can sponge directly on the mRNAs of lipid metabolism enzymes, which inhibit mRNA translation and reduce protein expression. MiR-320 can directly target fatty acid synthase (FASN) to restrain lung cancer growth via interfering with fatty acid metabolism [35]. MiR-4310 suppressed malignant progression of hepatoma by inhibiting FASN and SCD1-mediated lipid synthesis directly [36]. Some circRNAs were involved in regulating the activities of lipid metabolism-related enzymes, thus remodeling metabolic phenotypes and altering the processes of both the development and progression of cancer. CircFARSA, facilitated fatty acid synthesis and promoted lung adenocarcinoma progression via miR-330-5p/FASN axis [37]. The competitive binding of circRPL23A to miR-1233 directly targeted ACAT2 to restraining synthesis of cholesterol esters and inhibit the progression of renal carcinoma [16]. In the current studies, we explored the existence of hsa_circ_0021205/miR-195-5p/HSL axis, and verified its role in promoting malignant progression as well as enhancing both TAG and DAG lipolysis metabolism in glioblastoma, which disclosed that hsa_circ_0021205 acted as a miR-195-5p sponge, and miR-195-5p bound to the downstream target protein HSL, HSL was negatively correlated with the expression levels of miR-195-5p, and hsa_circ_0021205 negatively regulated miR-195-5p in glioblastoma, confirming the existence of hsa_circ_0021205 /miR-195-5p/HSL regulatory axis in glioblastoma development. Our results disclosed the regulatory pathway of circ_0021205/miR-195-5p/HSL on both aberrant lipid metabolism and EMT of glioblastoma. However, our findings were based on simple analysis of FA, MAG, DAG, and Oil red staining to evaluate lipid metabolism of tumor cells, which limited accurate interpretations of the regulatory role of the circ_0021205/miR-195-5p/HSL axis. Lipidomic analysis of both extracellular and intracellular lipids of tumor cells will help to fully elucidate the key mechanistic insights into the effect of the newly identified molecular regulatory axis.

MiR-195-5p has been identified as a tumor suppressor in osteosarcoma [38], colorectal cancer [39], and gliomas [40], which was consistent with our findings that overexpression of miR-195-5p can restrain proliferation, invasion, and migration of glioblastoma. For lack of a 5 ‘cap and 3’ polyadenylation tail, circRNAs can not form a traditional linear structure which can be cleaved by exonucleases, hence they had potential to serve as diagnostic and therapeutic biomarkers for tumor patients [41, 42]. Hsa_circ_0021205 expressed highly in cholangiocarcinoma and promoted tumor progression [43], while its roles in glioblastoma have not been investigated previously. Our studies suggested that knocking-down of hsa_circ_0021205 reduced the abilities of proliferation, invasion, and migration of glioblastoma cells in vitro, as well as in vivo glioblastoma growth. Although in vivo validations have verified the role of circ_0021205/miR-195-5p/HSL axis on regulating glioblastoma progression, the current preclinical glioblastoma models are limited by lack of natural tumor microenvironment and inability of most established tumor cell lines to accurately reproduce glioblastoma biology, which limited precise interpretations of the molecular regulatory axis. Patient-specific tumor tissue-derived cerebral organoids can help to further improve mimicry of human glioblastoma. Our preliminary investigations have shown that circ_0021205/miR-195-5p/HSL axis resulted in invasion and proliferation of glioblastoma cells, as well as promoting EMT. Although our data verified the correlation between the above-mentioned axis and phenotypic changes in tumor cells, the exact downstream signaling pathways activated by circ_0021205/miR-195-5p/HSL axis need further investigations, which can be explored through a proteomic or GSE approach, to fully elucidate the whole molecular regulatory pathways.

In general, the current studies identified HSL and its upstream regulator hsa_circ_0021205 as the potential biomarkers and regulators of glioblastoma. The hsa_circ_0021205/miR-195-5p/HSL axis played crucial roles in remodeling of glioblastoma abnormal lipolysis, and promoting EMT as well as glioblastoma progression, which provided new references in enhancing understanding of the regulatory role and mechanism of ncRNAs on glioblastoma lipid metabolism remodeling. However, the lipid homeostasis regulated by HSL is accommodated by complex feedback circuits, and the working mechanism of single regulatory pathway is not enough to elucidate all phenotypes of aberrant glioblastoma lipid metabolism. The transcription factors involved in the expression of HSL still need further investigations. For altering lipolysis may hinder other main metabolic processes, the current studies have not totally investigated the metabolic consequences of glioblastoma cells after interfering the identified circ_0021205/miR-195-5p/HSL axis. Panoramic profiling of other metabolic processes, such as glycolysis and mitochondrial respiration with bench bioanalyzers will be beneficial to elucidate regulatory mechanisms of the newly identified molecular axis at metabolomics level. Besides, the treatment strategy of glioblastoma via intervening lipolytic pathway is still in its infancy due to the diversity of key molecules regulating lipolysis in tumor cells [44, 45]. The current therapeutic schedule that targeted a key enzyme or a lipid metabolism pathway alone may have limited impact on suppressing tumor growth in clinical practice [46]. Previous studies have shown that glioblastoma cholesterol metabolism was related to remodeling of pro-tumor immunosuppressive microenvironment and the formation of high therapeutic resistance. Lipid metabolism remodeling profoundly affected the biological architectures of tumor microenvironment [47], promoted tumor progression and enhanced formation of tumor immunosuppressive microenvironment [48]. One of the features of glioblastoma is its high resistance to chemotherapy. In this respect, altered FA metabolism has been variously linked to therapy resistance of mesothelioma [49], cholangiocarcinoma [50]. Therefore, further exploring the translational relevance of the new molecular targets with chemo-resistance of glioblastoma will disclose the potential of the regulatory axis on regulating chemo-resistance of the glioblastoma, which may be a novel strategy against glioblastoma progression, but more in-depth mechanistic studies are still required.

## Conclusions

HSL was upregulated in glioblastoma and promoted tumor development via regulating the hsa_circ_0021205/miR-195-5p/HSL axis. This study revealed a new regulatory pathway targeting the lipolytic enzyme HSL in glioblastoma, which can help to further reveal the lipolytic network of glioblastoma and provide potential new therapeutic strategies against glioblastoma.

## Material and methods

### Cell lines and cell culture

Normal human astrocytes (NHAs) and human glioblastoma cell lines SNB19, LN229, U251MG, T98G and U87MG (Procell, Wuhan, China) were cultured in Dulbecco’s Modified Eagle’s Medium (DMEM) (Gibco, USA) supplemented with 10% fetal bovine serum (FBS) (Gibco, USA) at 37 °C in a humidified incubator with 5% CO_2_. Oleic acid (MCE, USA) was dissolved in fresh DMSO to 50 mM served as FAs stock solution, and further diluted with DMEM to make FAs rescue medium. Final concentrations of FAs were set 10 μM as working solution.

### Human clinical samples

From January 2019 to June 2021, 18 surgical specimens of glioblastoma patients and 8 paired peritumoral brain tissues were collected from the Second Affiliated Hospital of Soochow University (Suzhou, Jiangsu, China), and frozen in liquid nitrogen. Written informed consents were obtained from all patients and this study was approved by the Research Ethics Committee of the Second Affiliated Hospital of Soochow University (approval number: JD-LK-2018-027-02).

### Detection of lLipid-related molecules

Triglyceride assay kit (Abcam, USA) and free fatty acid assay kit (Abcam, USA) were utilized to detect intracellular triglyceride (TAG) and fatty acids (FAs) levels following the manufacturers’ protocols. Human MAG (Meibiao Biology, Jiangsu, China) and DAG ELISA (Meibiao Biology, Jiangsu, China) kits were applied to analyze intracellular level of monoglyceride (MAG) and diglyceride (DAG) according to the manufacturers’ protocols.

### Oil Red O staining

Cells were fixed with 1% paraformaldehyde for 30 min, then transferred to a freshly prepared staining mixture for 15 min (Beyotime, Shanghai, China). After washing with PBS for three times, cell nuclei were counterstained with hematoxylin for 1 min. Cells were observed and photographed under microscope (Zeiss, Germany).

### Transfection of siRNAs and plasmids

Small interfering RNAs (siRNAs) were synthesized by GenePharma (Suzhou, China). HSL overexpression plasmids were constructed based on pcDNA3.1vector (Genecreate, Wuhan, China). Cells were seeded in a 6-well plate and allowed to reach a confluence of 60–70% prior to transfection. Lipofectamine 2000 (Invitrogen, USA) was applied to transfect siRNAs into cells according to the manufacturer’s instructions. Subsequent experiments were performed 48 h post-transfection. The sequences of siRNAs are listed in Table S1.

### CCK-8 assay

Cells were seeded into a 96-well plate at a density of 2 × 10^3^ cells/well, 5 duplicate wells (100 μl DMEM per well) were set up, then placed in an incubator at 37 °C with 5% CO_2_. 10 μl of CCK-8 reagent (APExBIO, USA) was added in each well. After incubating at 37 °C for 1 h in the dark, the absorbance was detected at 450 nm wavelength with a microplate reader (Tecan, Switzerland) once a day for 4 consecutive days at the same time.

### Migration and invasion assays

DMEM diluted Matrigel (9:1) was coated on the bottom of the upper chamber surface of Transwell chamber (Costar, USA), and non-matrigel pre-coating only for the migration assay. Cell density was adjusted to 2.5 × 10^5^/ml in serum free medium, then 200 μL cell suspension was added to the upper chamber, and 500 μL medium containing 10% FBS to the lower chamber. After 48 h of cell culture at 37°C with 5% CO_2_, the matrigel and cells in the upper chamber were wiped off. All chambers were fixed with 4% paraformaldehyde for 20 min, and dyed with 0.5% crystal violet for 30 min. Cells were photographed under microscope.

### Colony formation

Cells were seeded in 6-well plate at a density of 500 cells/well in DMEM containing 10% FBS and cultured in an incubator at 37 °C with 5% CO_2_. After 12 days, cells were fixed with 1% paraformaldehyde for 30 min, then stained with 0.5% crystal violet for another 30 min.

### Wound healing assay

Cells were seeded in 6-well plate in DMEM contained in 10% FBS. After transfection of 48 h, 1.0 mL pipette tip was applied to draw a straight line on cell surface to create the wound surface. The living cells were maintained with FBS-free DMEM. Wound images were taken under a microscope (EVOS, USA) at 0 h, 24 h or 36 h, and the wound gap area was quantified with ImageJ.

### Quantitative real-time PCR (qRT-PCR)

Total RNA was extracted from cells with TRIzol (Invitrogen, USA), then reverse transcribed to cDNA with RevertAid First Strand cDNA Synthesis Kit (Thermo, USA). Quantitative RT-PCR was performed using SYBR Green (Vazyme, Nanjing, China) in QuantStudio 3 thermal cycler (ABI, USA). The relative expression level was calculated with 2^–ΔΔCt^ method. Actin (reference for circRNAs and mRNAs) and U6 (internal control for miRNAs) were utilized for normalization. The primers were synthesized by Sangon Biotechnology (Shanghai, China) and the relevant sequences are shown in Table S2.

### Western blot

Total cellular proteins were extracted with RIPA lysis buffer supplemented with protease and phosphatase inhibitors (Fudebio, Hangzhou, China). The concentration of total proteins was determined with a BCA kit (Thermo, USA). Total proteins (20 µg) were electrophoresed on 10% SDS-PAGE, then electro-transferred to PVDF membrane (Millipore, USA). The membrane was cut into strips and incubated individually with the primary antibodies at 4°C overnight, followed by incubation with the secondary antibodies for 1 h at room temperature. The bands were visualized with enhanced chemiluminescence system (Millipore, USA). The corresponding antibodies are listed in Table S3.

### Luciferase reporter assay

Binding sites for miR-195-5p with HSL (HSL-WT) or hsa_circ_0021205 (hsa_circ_0021205-WT) were predicted through online bioinformatic analysis, including Bioinformatics (https://www.bioinformatics.com.cn/) and Starbase 3.0 (https://starbase.sysu.edu.cn/). The potential binding sites for miR-195-5p with HSL (HSL-WT) or hsa_circ_0021205 (hsa_circ_0021205-WT), as well as the related mutated forms (HSL-MUT or hsa_circ_0021205-MUT) were inserted into the pmirGLO luciferase vector (Genecreate, Wuhan, China), respectively. SNB19 and LN229 cells were co-transfected with NC, miR-195-5p mimics and above-mentioned constructs (HSL-WT, HSL-MUT, hsa_circ_0021205-WT, hsa_circ_0021205-MUT), respectively. After 48 h, the luciferase activity of each sample was measured with Dual-Glo luciferase assay system (Promega, USA).

### Immunohistochemical staining

The clinical glioblastoma tissues and xenografts were fixed with 4% PFA and embedded in paraffin. Tissue slices were incubated with the indicated primary antibodies (shown in Table S3) at 4 °C overnight. After washing with PBS, sections were incubated with the biotinylated secondary antibodies at room temperature for 1 h, then were incubated with peroxidase solution for 30 min, followed by staining with DAB reagent (Roche, Switzerland) and counterstained with hematoxylin. The images of each section were taken and analyzed under an optical microscope.

### RNase R treatment

Total RNA extracted from SNB19 or LN229 cells was treated with RNase R (Yeasen, Shanghai, China) for 30 min at 37 °C according to the manufacturer’s instructions. The stability of hsa_circ_0021205 and WEE1 mRNA was analyzed by qRT-PCR.

### Actinomycin D treatment

Cells were cultured in six-well plate for 24 h, and fresh medium supplemented with 2 μg/ml actinomycin D (Sigma, USA) was added. Total cellular RNA was extracted at 0, 4, 8, 12 and 24 h respectively after actinomycin D supplementation for subsequent qRT-PCR analysis.

### Nucleocytoplasmic fractionation assay

Extraction and purification of cytoplasmic and nuclear RNAs were performed with Cytoplasmic & Nuclear RNA Purification Kit (Norgen, Canada) in accordance with the manufacturer’s protocols. Next, qRT-PCR was performed to quantify the expression of linear RNAs and circRNAs, with U6 and GAPDH as the internal references for nuclear and cytoplasmic RNAs, respectively.

### Stable transfection with lentiviral vector

Lentiviral expression of sh-NC, sh-hsa_circ_0021205, as well as the empty vector (EV) and HSL overexpression vector (OE) (Genepharma, Shanghai, China) were constructed, respectively. 48 h after transfection, positive cells were screened out with 5 µg/mL puromycin (MCE, USA) for 2 weeks to establish the stable transfection cell lines, and qRT-PCR were performed to evaluate transfection efficiency.

### In vivo xenograft model

All animal experiments were conducted in accordance with the ethical standards of the animal care and use committee of Soochow University (SUDA 20210708A03). Ectopic xenograft model was prepared by subcutaneous injection of 1 × 107 SNB19 cells (sh-NC, sh-hsa_circ_0021205, sh-hsa_circ_0021205 + HSL EV, or hsa_circ_0021205 + HSL OE) into the right armpit of 4-week-old female Balb/c nude mice (Sushang Biotechnology, Shanghai, China), four mice in each group. The tumor volume was measured and calculated by the formula *V* (mm^3^) = length × width^2^ × 0.5. The mice were sacrificed 6 weeks later under general anesthesia, and the xenografts were harvested. For evaluation of intracranial glioblastoma growth, orthotopic model was established. Briefly, a suspension of 1 × 105 SNB19 sh-NC or sh- hsa_circ_0021205 cells in 10 μl PBS was injected into the right caudate nucleus of the mice (randomly assigned into 3 mice/group). When neurological symptoms appeared obviously, mice were euthanized, then brains were sampled and fixed in 4% paraformaldehyde, followed by paraffin embedding and sectioning for hematoxylin and eosin staining.

### Bioinformatics analysis

MiRNA–mRNA interactions and the potential binding sites were predicted by starBase (http://starbase.sysu.edu.cn/) between miRNAs and HSL. The potential upstream target circRNAs of miR-195-5p were predicted based on bioinformatic analysis with Circbank (http://www.circbank.cn/). Microarray circRNA expression profile data of glioblastoma and corresponding peritumoral normal tissues were screened out from the Gene Expression Omnibus (GEO, https://www.ncbi.nlm.nih.gov/geo/) database. Differentially expressed circRNAs (DECs) in the GSE109569 datasets were analyzed and identified using GEO2R with the criteria of |log2 (fold change)| > 1 and *P* value < 0.05. The circRNAs upregulated in the datasets were selected, and intersected with the predicted circRNAs from Circbank.

### Statistical analysis

GraphPad Prism 9.0 software was used for statistical analysis of experimental data. All data was expressed as mean ± standard deviation. All experiments were repeated three times. The log-rank test was applied to compare survival differences between groups. Pearson’s correlation method was applied to analyze the correlation among HSL, miR-195-5p, hsa-circ-0021205. *P* < 0.05 was considered statistically significant (*p < 0.05; **p < 0.01; ***p < 0.001; ****p < 0.0001).

### Supplementary information

tables S1-S3

table S4

Original full length western blots

## Data Availability

All data generated or analyzed during this study are included in the article and its supplementary files, and available from the corresponding author on reasonable request.
