# Supplementary material for: Hsa_circ_0021205 enhances lipolysis via regulating miR-195-5p/HSL axis and drives malignant progression of glioblastoma
Source: Cell Death Discov. 2024 Feb 10;10:71. doi: 10.1038/s41420-024-01841-7 (PMC10858904; doi:10.1038/s41420-024-01841-7)
Supplement: Supplementary file 1 — tables S1-S3 [file 41420_2024_1841_MOESM1_ESM.docx]

**Table S1. The sequences of siRNAs were listed**

|  | **sense（5'-3'）** | **antisense（5'-3'）** |
| --- | --- | --- |
| **HSL#1** | 5'-GCAGCCUGAUAAAGUCCAATT-3' | 5'-UUGGACUUUAUCAGGCUGCTT-3 |
| **HSL#2** | 5'-GCAGGGAUCCAAUACCCAATT-3' | 5'-UUGGGUAUUGGAUCCCUGCTT-3' |
| **HSL#3** | 5'-GCAUAAGGGAUGCUUCUAUTT-3' | 5'-AUAGAAGCAUCCCUUAUGCTT-3' |
| **NC** | 5'-UUCUCCGAACGUGUCACGUTT-3' | 5'-ACGUGACACGUUCGGAGAATT-3' |
| **miR-195-5p mimics** | 5'-UAGCAGCACAGAAAUAUUGGC-3' | 5'-CAAUAUUUCUGUGCUGCUAUU-3' |
| **miR-195-5p inhibitor** | 5'-GCCAAUAUUUCUGUGCUGCUA-3' |  |
| **si-hsa-circ-0021205** | 5'- AAUUGUCUAAUUCCUGUGGTT-3' | 5'- CCACAGGAAUUAGACAAUUTT-3' |

**Table S2. The primers sequences were listed**

|  | Forward (5' to 3') | Reverse (5' to 3') |
| --- | --- | --- |
| HSL | 5'-cct cta ctc ctc acc cat agt c-3' | 5'-gtg gca ggc tct tga gca t-3' |
| miR-195-5p | 5'- cct cga gct agc agc aca gaa a-3' | 5'-atc cag tgc agg gtc cga gg-3' |
| hsa-circ-0021205 | 5'-ctg gta atg tcc ttc ccg ga-3' | 5'-tgg ctt cca tgt ctt cac ca-3' |
| U6 | 5'-ctc gct tcg gca gca ca-3' | 5'-aac gct tca cga att tgc gt -3' |
| β-actin | 5'-cct ggc acc cag cac aat-3' | 5'-ggg ccg gac tcg tca tac-3' |

miR-195-5p:RT primer 5'-gtc gta tcc agt gca ggg tcc gag gta ttc gca ctg gat acg acg cca at-3'

**Table S3. List of antibodies used in this study.**

| **Antigen** | **Cat. No** | **Company** | **Dilution** |
| --- | --- | --- | --- |
| **Antibodies for WB** |  |  |  |
| HSL | 17333-1-AP | Proteintech | 1:2000 |
| Anti-HSL(phospho S853) | ab109400 | Abcam | 1:1000 |
| Occludin | T55997F | Abmart | 1:1000 |
| N-Cadherin (D4R1H) | 13116 | CST | 1:1000 |
| Slug (C19G7) | 9585 | CST | 1:1000 |
| Beta-catenin(44C6) | M24002F | Abmart | 1:1000 |
| p-4E-BP1 (Thr37/46) | 2855 | CST | 1:1000 |
| 4E-BP1(53H11) | 9644 | CST | 1:1000 |
| Goat anti-Rabbit IgG (H+L) | 65-6120 | Invitrogen | 1:2000 |
| Goat anti-Mouse IgG (H+L) | A16066 | Invitrogen | 1:2000 |
| β-actin | 66009-1-Ig | Proteintech | 1:5000 |
| **Antibodies for IHC** |  |  |  |
| HSL | 17333-1-AP | Proteintech | 1:200 |
| Ki67 | 27309-1-AP | Proteintech | 1:200 |
