# Supplementary material for: Hsa_circ_0021205 enhances lipolysis via regulating miR-195-5p/HSL axis and drives malignant progression of glioblastoma
Source: Cell Death Discov. 2024 Feb 10;10:71. doi: 10.1038/s41420-024-01841-7 (PMC10858904; doi:10.1038/s41420-024-01841-7)
Supplement: Supplementary file 2 — table S4 [file 41420_2024_1841_MOESM2_ESM.docx]

Table S4. clinical characteristics of the patients

| number | age | Sex | position | surgical resection | concurrent radio-chemotherapy | Overall Survival, month |
| --- | --- | --- | --- | --- | --- | --- |
| 1 | 65 | woman | Lt frontal | total | yes | 21 |
| 2 | 66 | man | Lt frontal | total | yes | 24 |
| 3 | 61 | woman | Rt frontal-insular | total | yes | 18 |
| 4 | 66 | man | Rt tempo-occipital | total | yes | 19 |
| 5 | 71 | woman | Lt frontal-callosum | subtotal | yes | 12 |
| 6 | 59 | man | Rt temporal | total | yes | 29 |
| 7 | 63 | woman | Lt frontal-tempo | total | yes | 12 |
| 8 | 71 | man | Lt frontal-tempo | subtotal | yes | 13 |
| 9 | 68 | woman | Rt temporal | total | yes | 27 |
| 10 | 78 | man | Lt temporal | total | yes | 18 |
| 11 | 77 | man | Rt parietal-occipital | total | yes | 21 |
| 12 | 69 | man | Rt temporal-parietal | subtotal | yes | 11 |
| 13 | 57 | man | Lt frontal-parietal | subtotal | yes | 10 |
| 14 | 60 | man | Lt frontal | total | yes | 23 |
| 15 | 71 | man | Lt frontal-tempo | subtotal | yes | 16 |
| 16 | 53 | woman | Rt frontal | total | yes | 19 |
| 17 | 87 | woman | Rt temporal | total | yes | 19 |
| 18 | 56 | woman | Lt frontal | total | yes | 22 |
