# Supplementary material for: Hsa_circ_0021205 enhances lipolysis via regulating miR-195-5p/HSL axis and drives malignant progression of glioblastoma
Source: Cell Death Discov. 2024 Feb 10;10:71. doi: 10.1038/s41420-024-01841-7 (PMC10858904; doi:10.1038/s41420-024-01841-7)
Supplement: Supplementary file 3 — Original full length western blots [file 41420_2024_1841_MOESM3_ESM.pdf]

Figure 1B

HSL
100 kDa

β-actin
42 kDa

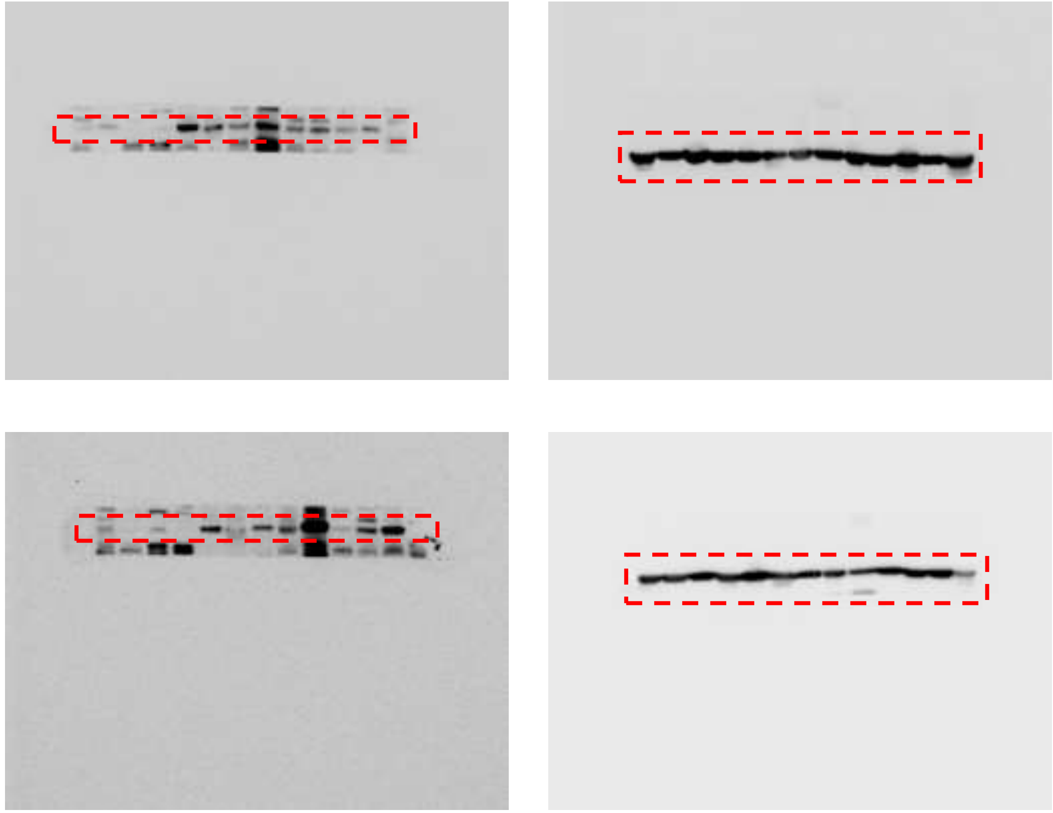

Figure 1F

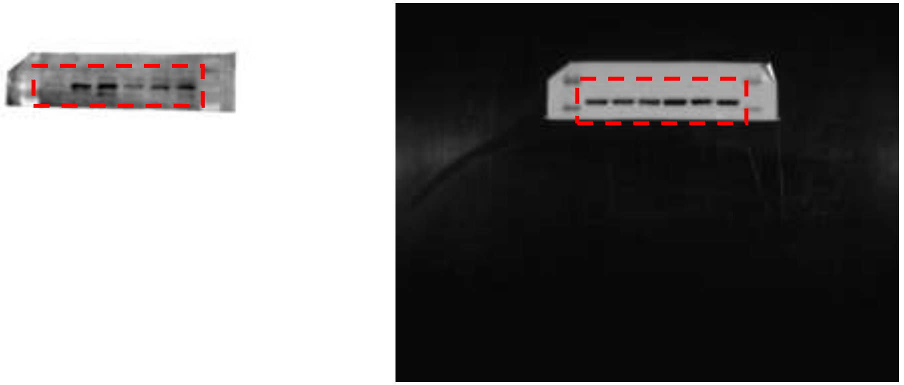

Figure 2B

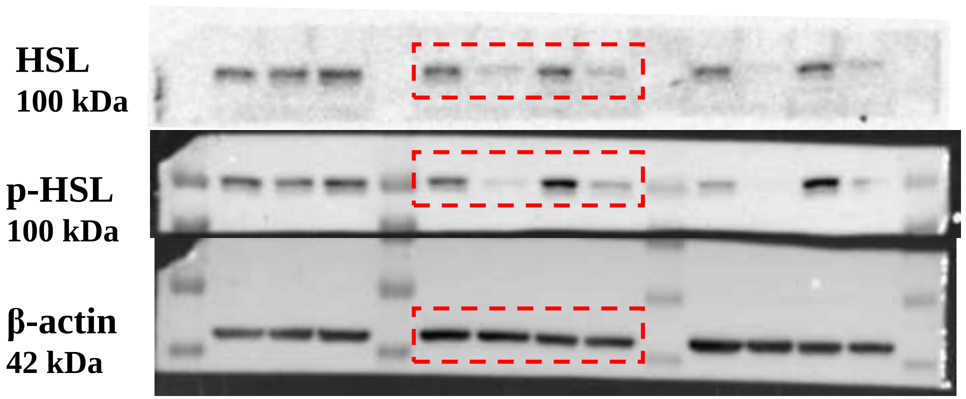

Figure 3B

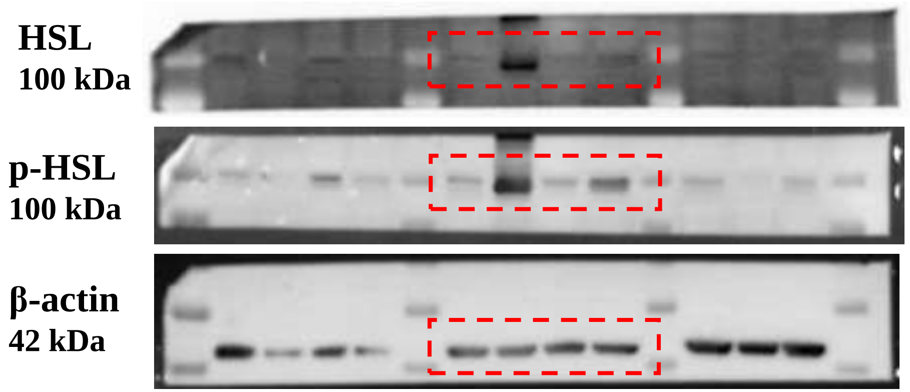

SNB19

Figure 2K

N-cadherin 140kDa

p-HSL 100 kDa

Occludin 59 kDa

β-actin 42 kDa

Slug 30 kDa

P-4E-BP1 15 kDa

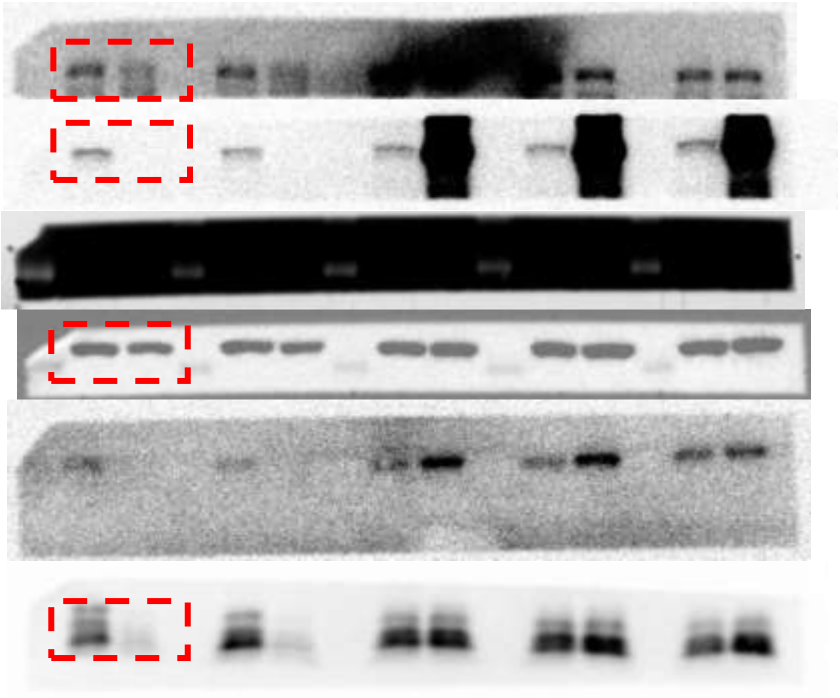

Figure 2K

p-HSL 100 kDa

Occludin 59 kDa

β-actin 42 kDa

Slug 30 kDa

4E-BP1 15 kDa

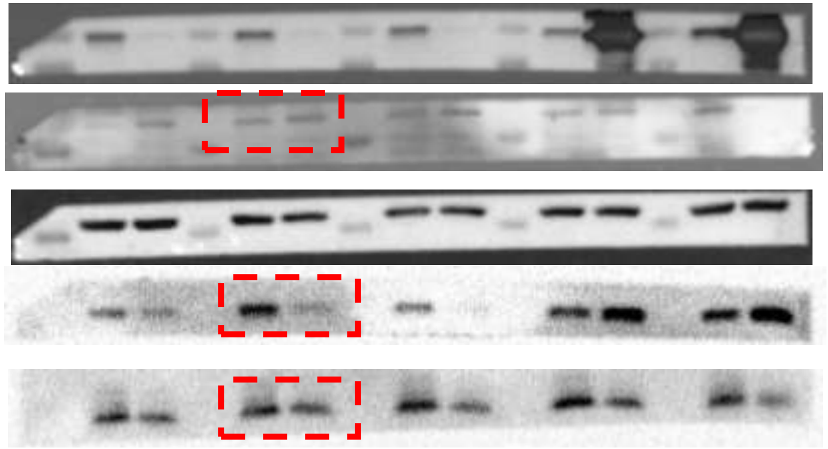

SNB19

Figure 2K

β-catenin 92 kDa

β-actin 42 kDa

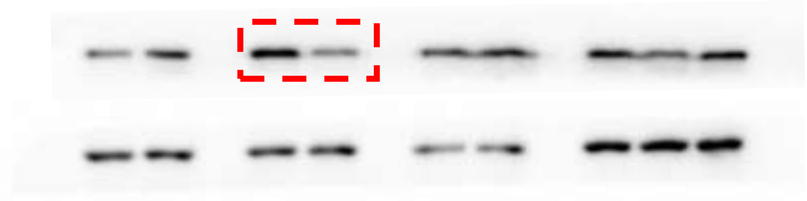

LN229

Figure 2K

β-catenin 92 kDa
p-HSL 100 kDa
Occludin 59 kDa
β-actin 42 kDa
Slug 30 kDa
P-4E-BP1 15 kDa
4E-BP1 15 kDa

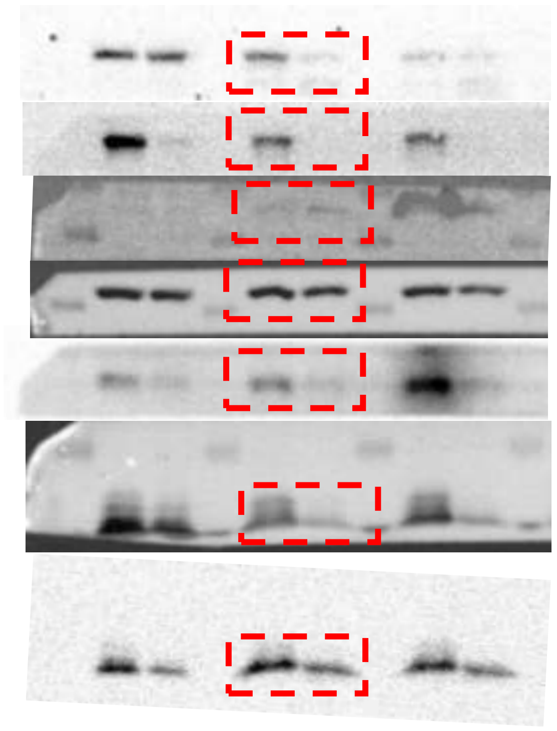

Figure 3K **Figure 2K**

N-cadherin 140kDa
p-HSL 100 kDa
Occludin 59 kDa
β-actin 42 kDa
Slug 30 kDa

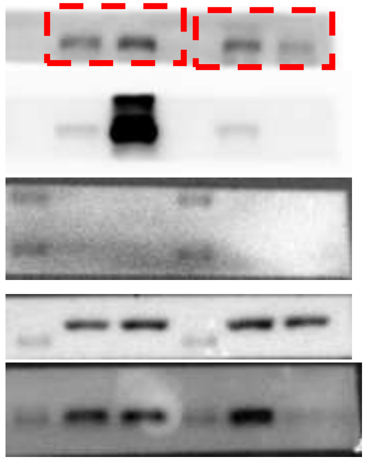

LN229

Figure 3K

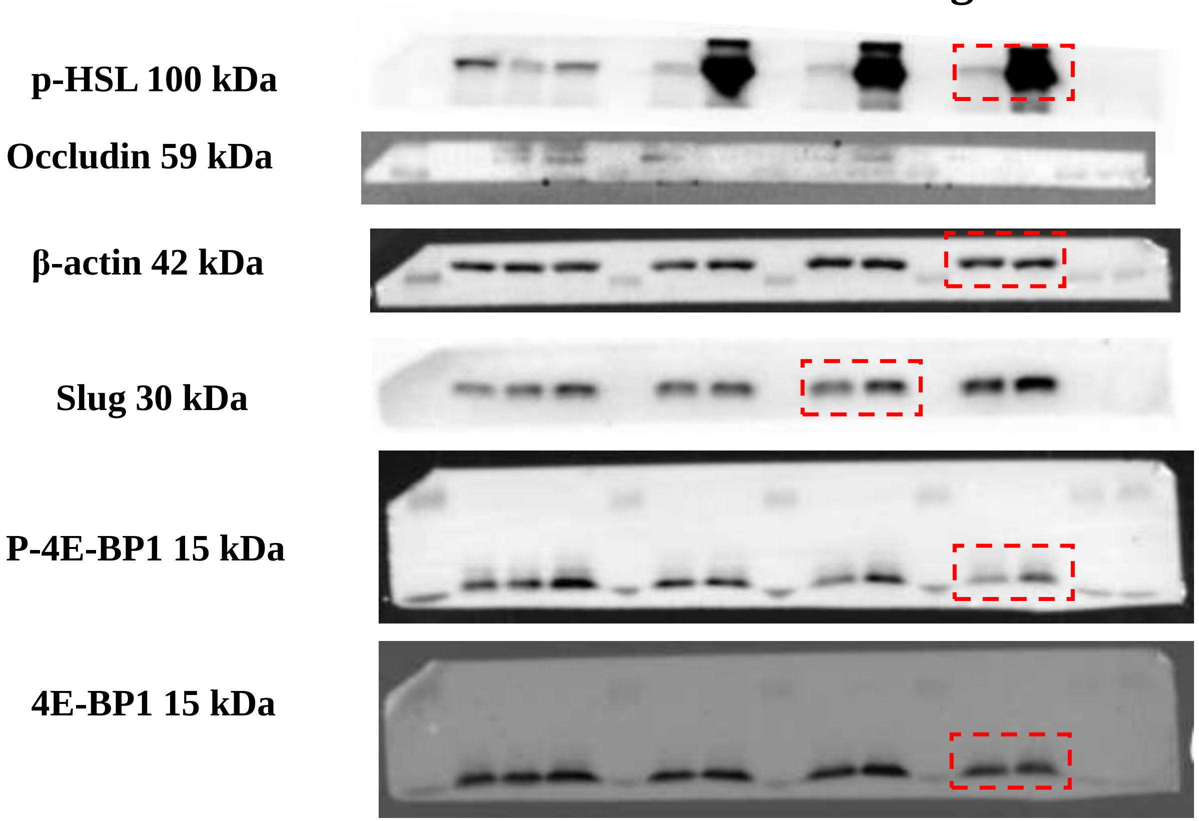

Figure 3K

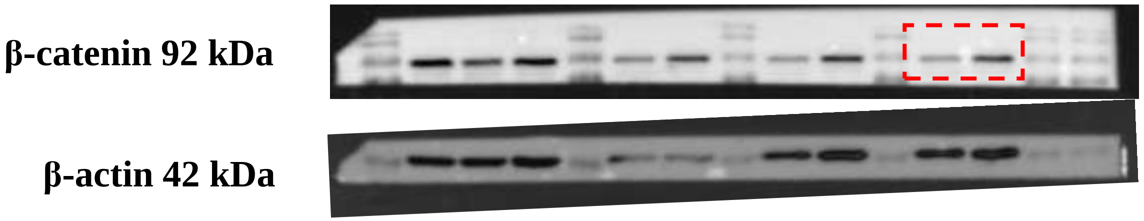

Figure 3K Figure 3K

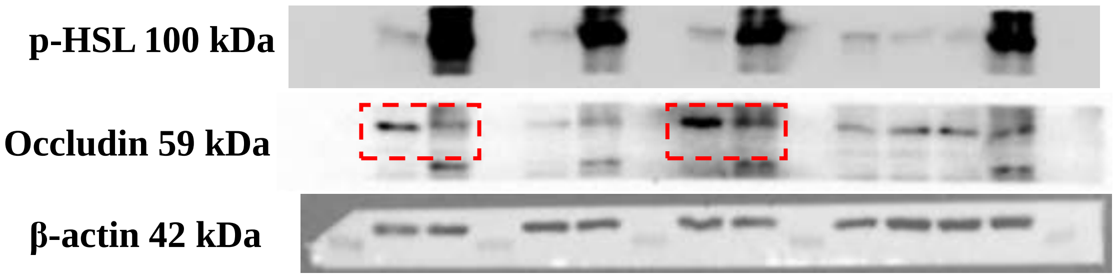

Figure 4I

snb19

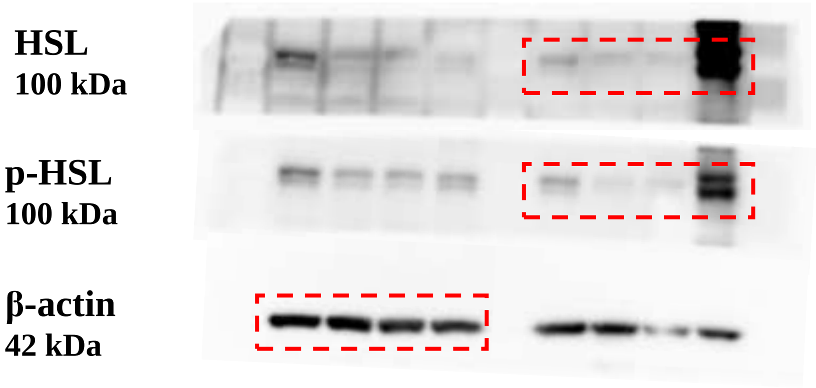

Figure 4I

LN229

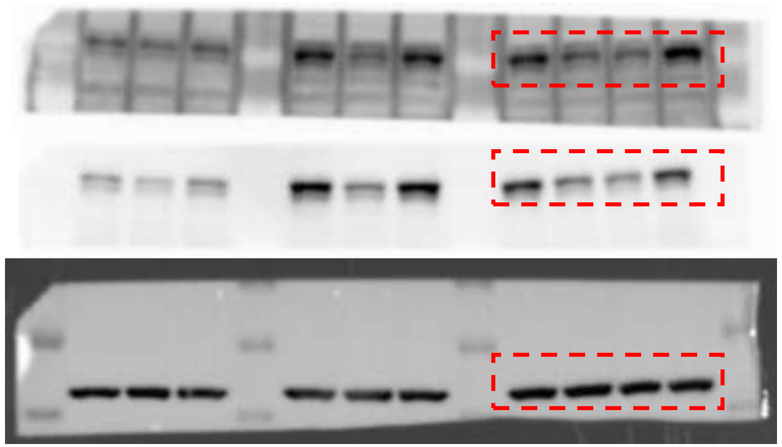

Figure 6G

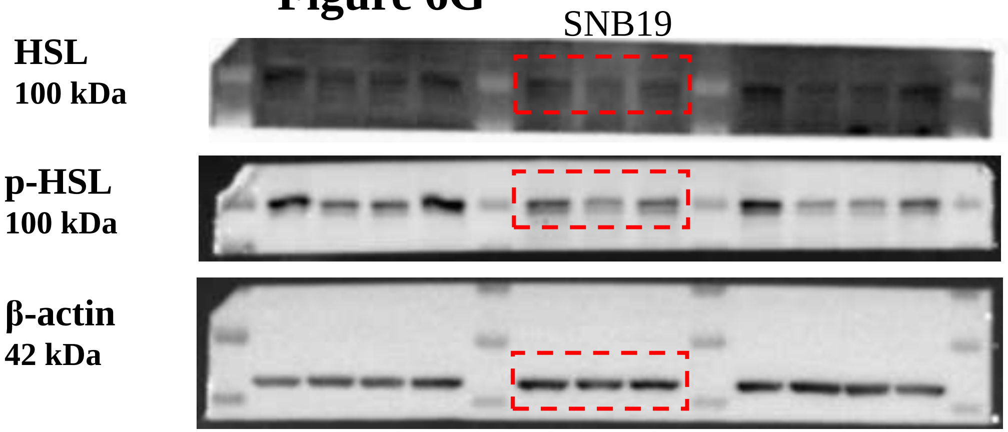

Figure 6G

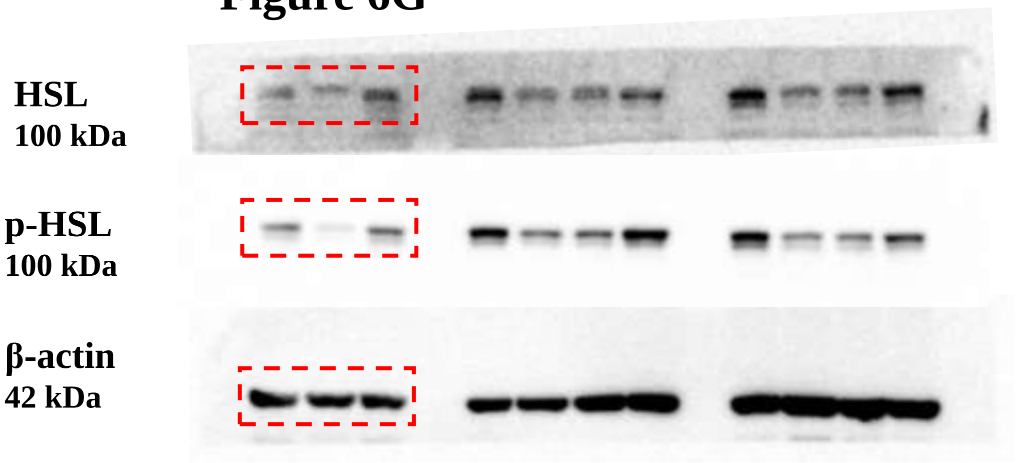

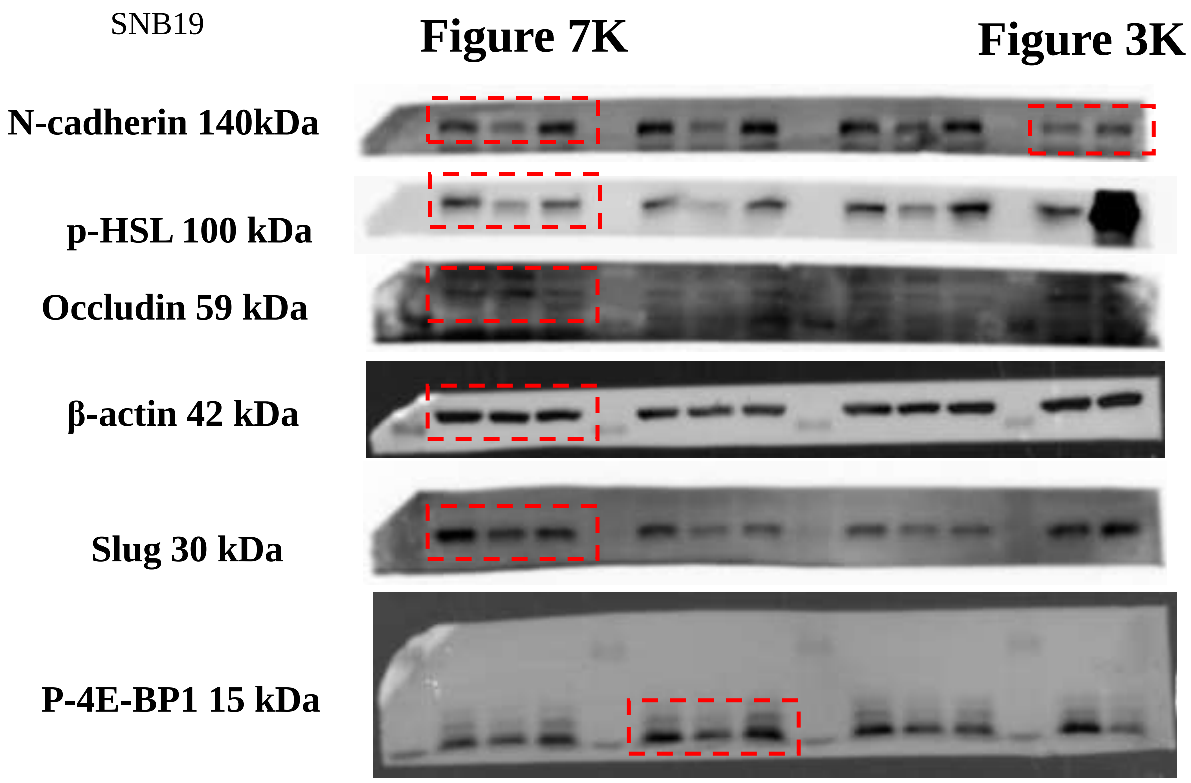
SNB19
Figure 7K
Figure 3K
N-cadherin 140kDa
p-HSL 100 kDa
Occludin 59 kDa
β-actin 42 kDa
Slug 30 kDa
P-4E-BP1 15 kDa

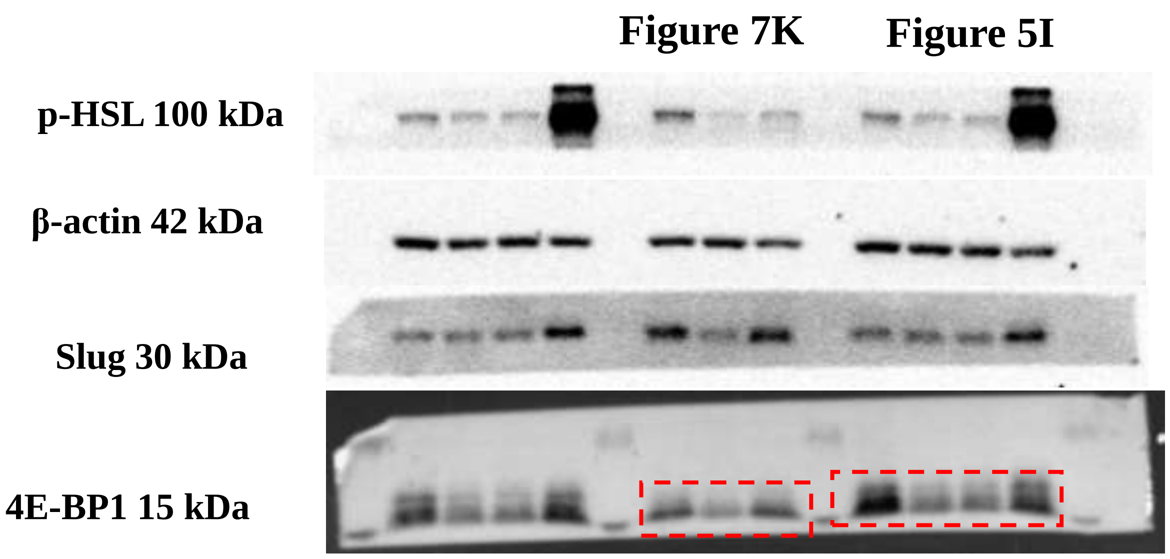
Figure 7K
Figure 5I
p-HSL 100 kDa
β-actin 42 kDa
Slug 30 kDa
4E-BP1 15 kDa

SNB19

Figure 7K Figure 5I

N-cadherin 140kDa
β-catenin 92 kDa
p-HSL 100 kDa
Occludin 59 kDa
β-actin 42 kDa
Slug 30 kDa
P-4E-BP1 15 kDa

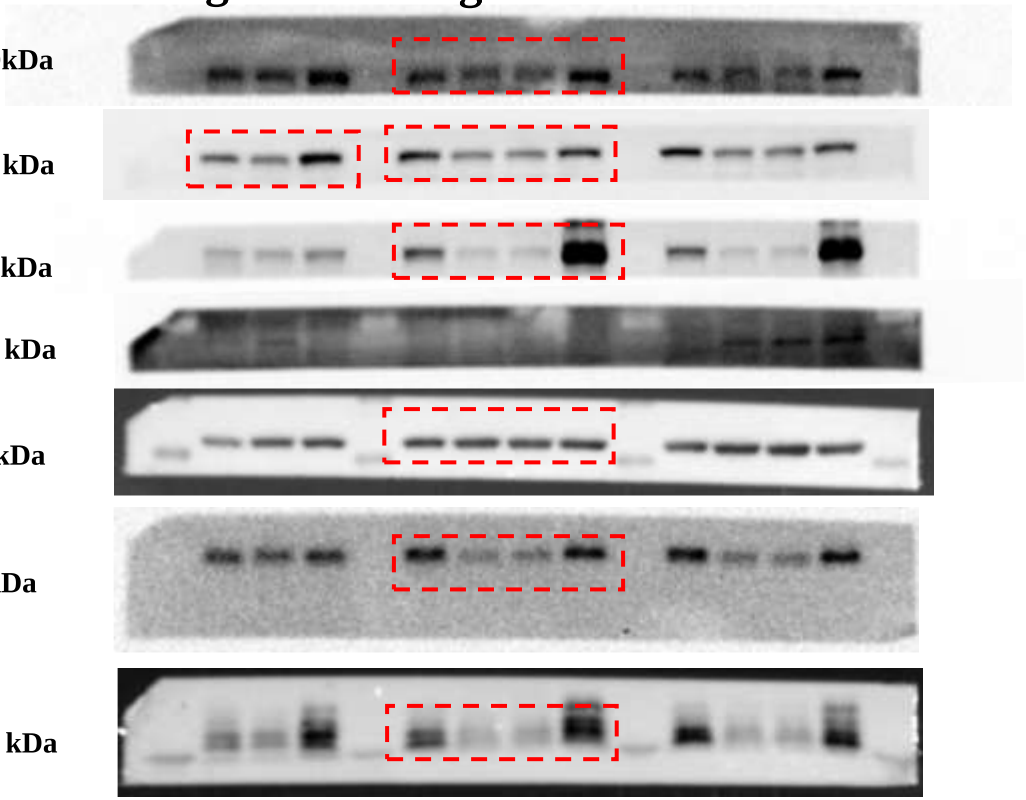

Figure 3K

N-cadherin 140kDa
β-catenin 92 kDa
p-HSL 100 kDa
Occludin 59 kDa
β-actin 42 kDa
Slug 30 kDa
P-4E-BP1 15 kDa
4E-BP1 15 kDa

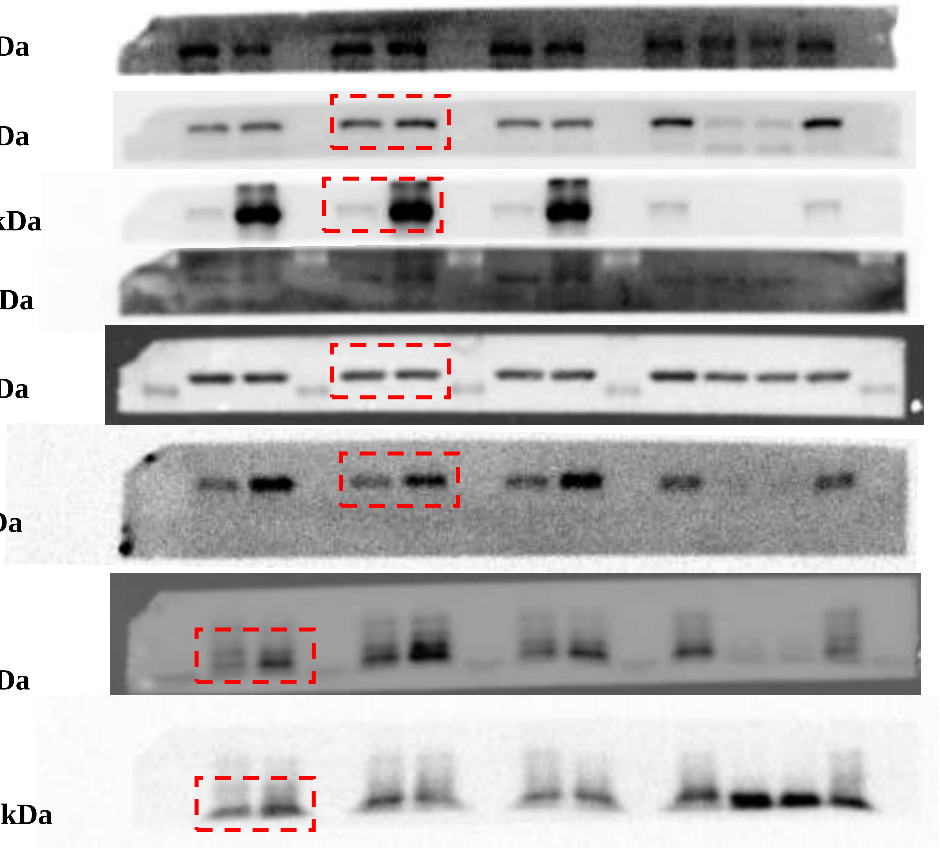

Figure 5I

p-HSL 100 kDa
Occludin 59 kDa
β-actin 42 kDa

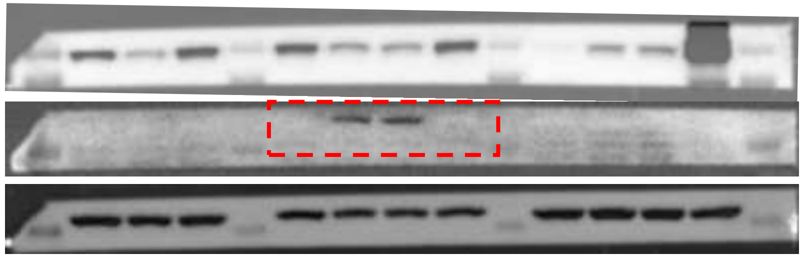

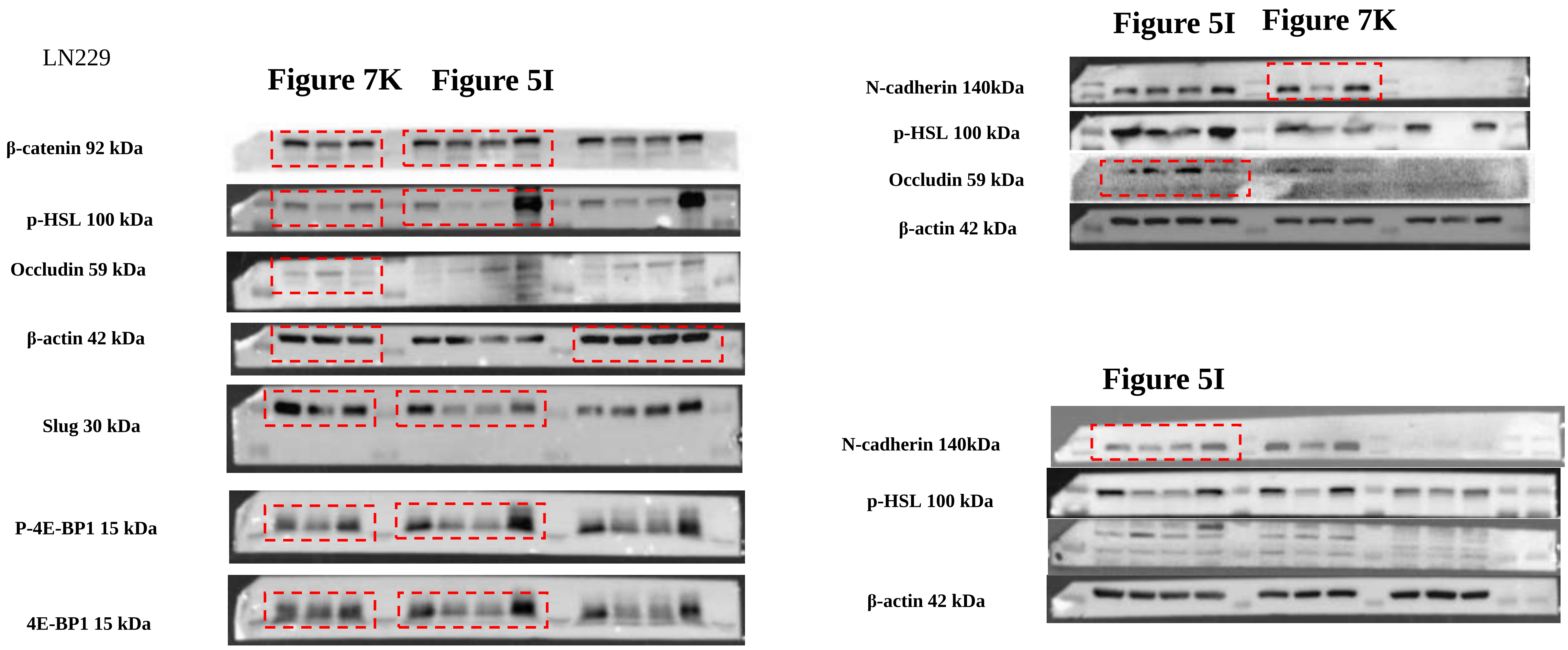
LN229
Figure 7K
Figure 5I
β-catenin 92 kDa
p-HSL 100 kDa
Occludin 59 kDa
β-actin 42 kDa
Slug 30 kDa
P-4E-BP1 15 kDa
4E-BP1 15 kDa
Figure 5I
Figure 7K
N-cadherin 140kDa
p-HSL 100 kDa
Occludin 59 kDa
β-actin 42 kDa
Figure 5I
N-cadherin 140kDa
p-HSL 100 kDa
β-actin 42 kDa
